# The effect of discrepancy between radiologic size and pathologic tumor size in renal cell cancer

**DOI:** 10.1186/s40064-016-2645-z

**Published:** 2016-06-27

**Authors:** Ning Zhang, Yishuo Wu, Jianqing Wang, Jianfeng Xu, Rong Na, Xiang Wang

**Affiliations:** Department of Urology, Huashan Hospital, Fudan University, No. 12 Central Urumchi Road, Shanghai, 200040 People’s Republic of China; Fudan Institute of Urology, Huashan Hospital, Fudan University, No. 12 Central Urumchi Road, Shanghai, 200040 People’s Republic of China

**Keywords:** Renal cell cancer, Nephrectomy, Tumor size, Radiologic, Pathologic

## Abstract

To investigate the difference between preoperative radiologic tumor size (RTS) and postoperative pathologic tumor size (PTS) in patients who underwent nephrectomy for renal cell carcinoma. We retrospectively reviewed 257 patients who received preoperative computed tomography (CT) before radical or partial nephrectomy for renal cell carcinoma from January 2010 to May 2015 in Huashan Hospital, Shanghai. RTS was defined as the largest diameter of tumor measured by CT and PTS as the largest diameter of tumor measured in the surgical specimens. Among all subjects, mean RTS was larger than PTS (4.57 ± 2.15 vs. 4.02 ± 2.15 cm, *P* = 0.004) with a discrepancy of 0.55 cm. When the patients were categorized according to T stage, the mean RTS was greater than PTS in the following groups: ≤4 cm group (2.90 vs. 2.59 cm, *P* = 0.02), >4 and ≤7 cm group (5.08 vs. 4.38 cm, *P* < 0.0001), except for >7 cm (8.9 vs. 8.0 cm, *P* = 0.142). Among patients with clear cell RCC, the mean RTS was larger than the mean PTS (4.57 vs. 3.98 cm, *P* = 0.004), similar result was also seen in non-clear cell group (4.54 vs. 4.16 cm, *P* = 0.045). The mean RTS was larger than PTS for the approach of radical nephrectomy (RN) (5.26 vs. 4.64 cm, *P* = 0.01), but not for the partial nephrectomy (PN) (3.34 vs. 2.92 cm, *P* = 0.067). Of the 257 renal cancers, 76 tumors were down-staged when comparing radiographic and pathologic tumor maximal diameter. The proportion of down-staged tumors had no difference between different genders (*P* = 0.283), different surgery approaches (*P* = 0.102), and different pathology types (*P* = 0.209). In this study, we found that renal tumor size was overestimated by radiography compared with pathologic results, and the T staging of some tumors was down-staged. But for patients who underwent PN, there was no difference between RTS and PTS. These results suggested that the PN should be considered first for the T1b renal tumor when tumor size was close to 4 cm, while the recommendation level of PN for T1b tumor was grade B according to EAU guidelines.

## Background

Renal cell cancer (RCC) represents 2–3 % of all cancers. The estimated new cases and deaths were 61,560 and 14,080 in US in 2015 (Siegel et al. 2015). Despite the rapid increase for several decades, the incidence rates of RCC stabilized during the year of 2007 and 2011. It may partially attribute to the increasing use of abdominal imaging test in annual heath examination. In addition, the death rates decreased by 0.9 % per year from 2007 to 2011 (American Cancer Society 2015). Nevertheless, due to the relatively high incidence, RCC had became one of the most important healthcare issues worldwide.

For the localised RCC, surgery is the only curative treatment with high-quality evidence. Partial nephrectomy (PN) and radical nephrectomy (RN) are the two major styles of surgical procedures. And the size of a renal tumor is important for staging, prognosis and the selection of the appropriate surgical procedure. For localized tumor, of which the T staging is T1, PN is recommended by guidelines (Motzer et al. 2015; Ljungberg et al. 2015). The decision of performing PN is normally determined by the radiologic size, but not the pathologic size. The radiologic size of tumor is usually measured by preoperative CT scan (Satasivam et al. 2012). Therefore, it is necessary to investigate the difference between pathologic and radiologic sizes, which would help urologists to make better decisions in clinical practice.

Some studies have revealed that there existed a certain degree of discrepancy between the preoperative size of renal tumors as measured by CT and the pathologic size as determined from surgical specimens (Choi et al. 2015; Chen et al. 2013; Lee et al. 2010). Since a discrepancy often exists between the preoperative radiologic tumor size (RTS) and the postoperative pathologic tumor size (PTS), the over-estimated tumor size by CT might cause the upstage of preoperative T stage and lead to the loss of opportunity to receive PN for quite a number of the patients (Kanofsky et al. 2006; Aertsen et al. 2013). Thus we performed this study to evaluate whether the discrepancies between the radiologic and pathologic sizes have an impact on tumor staging and the appropriate choice of surgical procedure.

## Results

In current study, a total of 257 patients were included, among which 181 were men (70.4 %) and 76 were women (29.6 %). The baseline characteristics of the patients were shown in Table 1. The median age was 56.8 years (range 18–86 years) and the median BMI was 24.46 (range 15.63–32.37). Among these subjects, 164 (63.8 %) received RN and 96 (36.2 %) underwent PN. Among all the patients, there were 183 patients (71.2 %) with T1a clinical stage and 57 (22.2 %) with T1b clinical stage. The most common histologic subtype was clear cell (80.9 %). All tumors had no positive margins.

**Table 1:** Demography

Feature	Median ± SD or n (%)	RTS (cm)	PTS (cm)	*P* value
No. of total subjects	257			
Age (years)	56.8 (range 18–86)			
Gender				
Male	181 (70.4 %)	4.41 ± 1.96	3.84 ± 1.85	<0.0001
Female	76 (29.6 %)	4.93 ± 2.54	4.43 ± 2.72	<0.0001
BMI	24.46 (range 15.63–32.37)			
Tumor side				
Left	117 (45.5 %)	4.70 ± 2.25	4.16 ± 2.28	<0.0001
Right	140 (54.5 %)	4.46 ± 2.07	3.90 ± 2.04	<0.0001
Surgery type				
RN	164 (63.8 %)	5.26 ± 2.12	4.64 ± 2.21	0.01
PN	93 (36.2 %)	3.34 ± 1.60	2.92 ± 1.54	0.067
Histology				
Clear cell	208 (80.9 %)	4.57 ± 2.09	3.98 ± 2.06	0.004
Chromophobe	9 (3.5 %)	4.54 ± 2.42	4.16 ± 2.51	0.045
Papillary	11 (4.3 %)			
Collecting duct	1 (0.4 %)			
Adenocarcinoma	2 (0.8 %)			
ccRCC + adenocarcinoma	5 (1.9 %)			
Other	21 (8.2 %)			
Pathologic T stage				
T1a	183 (71.2 %)	3.67 ± 1.25	3.04 ± 0.97	<0.0001
T1b	57 (22.2 %)	5.87 ± 1.31	5.37 ± 0.87	0.001
T2a	11 (4.3 %)			
T2b	6 (2.3 %)			
RTS (cm)	4.57 ± 2.15			0.004
PTS (cm)	4.02 ± 2.15			

The mean RTS were larger than PTS (4.57 ± 2.15 vs. 4.02 ± 2.15 cm, *P* = 0.004) with a discrepancy of 0.55 cm. In addition, when the RTS was ≤4 cm, the mean RTS (2.90 cm) was still larger than PTS (2.59 cm) (*P* = 0.002) and such difference also existed when the RTS was 4–7 cm (RTS 5.08 cm vs. PTS 4.38 cm, *P* < 0.0001). However, when the RTS was > 7 cm, the mean RTS (8.9 cm) and mean PTS (8.0 cm) were not statistically different (*P* = 0.142) (Table 2).

**Table 2:** The mean RTS and PTS by RTS and clinical stage

T-stage	RS range	N	RTS (cm)	PTS (cm)	*P* value
T1a	≤4 cm	115 (44.7 %)	2.90 ± 0.73	2.59 ± 0.76	0.002
T1b	>4, ≤7 cm	111 (43.2 %)	5.08 ± 0.82	4.38 ± 1.19	<0.0001
T2	>7 cm	31 (12.1 %)	8.9 ± 0.73	8.0 ± 2.73	0.142
	Total	257	4.57 ± 2.15	4.02 ± 2.15	0.004

In subgroup analysis, among patients with clear cell RCC, the mean RTS was larger than the mean PTS (4.57 vs. 3.98 cm, *P* = 0.004), and same for the non-clear cell group (4.54 vs. 4.16 cm, *P* = 0.045). The mean RTS was significantly larger than PTS (5.26 vs. 4.64 cm, *P* = 0.01) for the approach of RN, but not for the approach of PN (3.34 vs. 2.92 cm, *P* = 0.067).

Of the 257 renal cancers, 76 tumors were down-staged when comparing radiographic and pathologic tumor maximal diameter including 62 T1b → T1a (21.4 %) and 14 T2a → T1b (8.2 %). And there was significantly difference (*P* < 0.0001) between RTS and PTS When the patients were categorized by down-staged tumors (Table 3). Besides, there was no difference of down-staged tumor proportion (*P* = 0.283) between male and female, PN and RN (*P* = 0.102), or ccRCC and non-ccRCC (*P* = 0.209).

**Table 3:** The mean RTS and PTS by down-staged tumors

Down-staged tumors	N	RTS (cm)	PTS (cm)	*P* value
T1b → T1a	62 (21.4 %)	4.74 ± 0.64	3.60 ± 0.50	<0.0001
T2a → T1b	14 (8.2 %)	7.54 ± 0.33	6.00 ± 0.81	<0.0001
No change	181 (70.4 %)	4.28 ± 2.38	4.01 ± 2.47	<0.0001

## Discussion

In this study, we found that radiologically measured size of renal mass was overestimated compared with pathologic size as previously reported by others. And there was statistically difference between RTS and PTS for different tumor side and gender. Besides, the T staging of some tumors was down-staged. However, observed discrepancy between the preoperative size of renal tumors as measured by CT and the pathologic size as determined from surgical specimens was minimal, usually less than 1 cm, which was statistically insignificant. And for the result of PN procedure, there was no difference between RTS and PTS. According to our results, we thought that CT generally provided an accurate assessment of the actual size of renal tumors. We considered that PN should be considered first for the T1b tumor when its size was close to 4 cm, while the recommendation for PN of T1b was grade B according to EAU guidelines. Although the difference between RTS and PTS was statistically significant, we do not think this disparity represents a clinically significant result.

PN surgery is recommended (Grade A) in patients with T1a tumors according to EAU guideline (Ljungberg et al. 2015). But more recent researches suggested that a threshold of >4 cm and even 7 cm for appropriately selected patients was safe and effective (Roos et al. 2011; Becker et al. 2011). In our study, the tumors with radiographic size close to 4 cm were overestimated by CT, and such discrepancy might affect the choice between RN and PN. However, the situation is different if the size of the tumor was larger than 4 cm. In some centers, a tumor size of 4 cm is still regarded as the cutoff between RN and PN. Recent studies have shown that PN for renal tumors had superior intermediate-term preservation of renal function, and similar recurrence rate compared with RN (Favaretto et al. 2013; Lane et al. 2010). Our results suggested that the PN should be considered first for the T1b tumor whose size was close to 4 cm, namely the threshold of tumor size of 4 cm for PN should been expanded to some extent.

A reduction in renal tumor size is commonly observed after surgical resection because of a loss of blood in the tumor. This tumor size reduction has an impact on the final pathologic stage in organ-confined tumors for which size is the only criterion. There were still some limitations in the current study. (1), this study was retrospective, and patients were from a single institution, which might result in the bias commonly seen in such kind of studies. (2), the sample size was relatively small, and we needed more cases to validate our results (3), a sub-analysis was not performed for non-clear cell histology due to the relatively small number of cases, and the data of Fuhrman grading was lacking.

## Conclusions

In this study, we found that renal tumor size was overestimated by radiography compared with pathologic results, and the T staging of some tumors was down-staged. But for patients who underwent PN, there was no difference between RTS and PTS. These results suggested that the PN should be considered first for the T1b renal tumor when tumor size was close to 4 cm, while the recommendation level of PN for T1b tumor was grade B according to EAU guidelines.

## Methods

The records of 257 patients from Huashan Hospital, Shanghai, who underwent PN or RN for renal tumors from January 2010 to May 2015 were retrospectively reviewed upon receiving the approval from our institutional review board. All the patients were undergoing preoperative CT scans at our institution within 4 weeks before surgery. RTS was defined as the largest diameter of tumor measured by CT images and PTS was defined as the largest diameter of tumor measured in the surgical specimen. We compared and analyzed the two parameters. Other clinical information, including patients’ age, gender, body mass index (BMI), histologic subtype of tumor, tumor side, tumor T staging, and type of surgery received, were also assessed. The tumors were staged radiologically and pathologically using the 2010 TNM staging system as follows: T1a, less than 4 cm; T1b, 4 cm or more but less than 7 cm; and T2, larger than 7 cm. Downstaging of tumors was determined by comparing the radiologic stage with the pathologic stage.

In our analysis, patients were categorized according to patients’ age, gender, histologic subtype, tumor side, T staging, operating approach and down-staged tumors. Mean values of radiological and pathologic tumor size along with differences in radiographic and pathologic tumor sizes were calculated for each category. The SPSS software package version 11.0 (Statistical Package for Social Sciences, Chicago, IL, USA) was used for statistical analysis. Data for Continuous variables were shown as median ± standard deviation and compared using the Student’s *t* test. Categorical variables were shown as percentage and compared using Pearson’s Chi square test. The comparison between tumor sizes was performed using the paired *t* test. A *P* value of <0.05 was considered to be statistically significant.

## Abbreviations

RCC: renal cell cancer
PN: partial nephrectomy
RN: radical nephrectomy
RTS: radiologic tumor size
PTS: pathologic tumor size
CT: computed tomography
